# Taxane-associated retinopathy and radiation-induced optic neuropathy in a young female patient with metastatic breast cancer

**DOI:** 10.3205/oc000255

**Published:** 2025-07-15

**Authors:** Suher Abduraman, Bogdana Maliş, Ali Riza Cenk Celebi

**Affiliations:** 1 Clinical Emergency Eye Hospital, Bucharest, Romania; 2 Department of Ophthalmology, Acibadem University School of Medicine, Istanbul, Turkey

**Keywords:** breast cancer, paclitaxel, hydroxychloroquine, central serous chorioretinopathy, macular edema, radiation-induced optic neuropathy

## Abstract

**Introduction:**

Metastatic breast cancer leads to significant challenges in terms of treatment and management, often requiring a multidisciplinary approach due to the potential side effects of chemotherapy and radiotherapy.

**Case description:**

We present a case of a metastatic breast cancer patient with central serous chorioretinopathy, recurrent cystoid macular edema, and radiation-induced optic neuropathy that occurred after the treatment with paclitaxel and radiation. A 42-year-old female patient presented to our department with a one-week history of painless, subacute vision loss in her left eye, occurring one year after completing oncological treatment. Her best corrected visual acuity (BCVA) was 20/32 in her left eye, and the optical coherence tomography (OCT) showed central serous chorioretinopathy with spontaneous resolution within 1 month. Seven months later, she presented with a sudden decrease in vision in her right eye; the BCVA was 20/40, the relative afferent pupillary defect was found, and the visual field demonstrated a superior altitudinal defect corresponding to the inferior segmental optic nerve pallor, prompting us to start treatment with prednisolone. Six months later, her vision had fallen to light perception in the right eye and 20/25 in the left eye. The OCT findings were conclusive for cystoid macular edema in her left eye, so we started treatment with aflibercept. Unfortunately, we could not improve the visual outcome in the right eye, which had an amaurotic pupil. Regarding the left eye, the patient experienced recurrent macular edema treated with aflibercept. After several episodes, the patient’s BCVA decreased to 20/50 without any improvement.

**Conclusion:**

Herein we stated a young patient with metastatic breast cancer who developed a rare and unusual overlap of side effects: paclitaxel-associated retinopathy and radiation-induced optic neuropathy. We aim to illustrate the challenge of managing advanced breast cancer patients and emphasize the importance of careful monitoring for ocular complications, which can impact the patient’s quality of life.

## Introduction

Breast cancer is the most prevalent cancer among women, continuing to be a significant health problem worldwide. Treatment options include a combination of surgery, chemotherapy, radiation, hormone therapy, and targeted therapies. Managing metastatic breast cancer involves navigating complex therapeutic challenges, including the development of drug resistance and the balancing of treatment efficacy against toxicity. Patients often require radiation and chemotherapy, resulting in compounded side effects that can impact their quality of life [1].

Paclitaxel is known as the first microtubule-stabilizing agent from the taxane family and is used in many malignancies because of its antitumoral effect [2]. Ophthalmic side effects caused by taxanes are rare, but include meibomian gland dysfunction, cystoid macular edema, canalicular obstruction, diplopia, lash alopecia, blepharitis, chalazion, eyelid edema, anisocoria, photopsia, cranial nerve six palsy and central retinal artery occlusion [3]. However, there is limited information available to accurately estimate how often various ophthalmic side effects occur in patients undergoing taxane therapy for systemic cancers. 

Radiation-induced optic neuropathy (RION) is also a rare complication that can occur when the optic nerves are exposed to radiation during radiotherapy. It presents as sudden, painless vision loss in one eye, typically occurring long after exposure to radiation. Diagnosis is primarily based on excluding other conditions and analyzing the clinical history in relation to the timing of radiation exposure. Treatment options are limited and generally ineffective [4]. 

This case report highlights a rare and unusual overlap of side effects: paclitaxel-associated retinopathy and radiation-induced optic neuropathy in a young female patient with metastatic breast cancer. We aim to illustrate the complexities of managing advanced breast cancer patients and emphasize the importance of careful monitoring for ocular complications, which can profoundly affect the patient’s quality of life and overall treatment experience. 

## Case description

**Figure 1:**
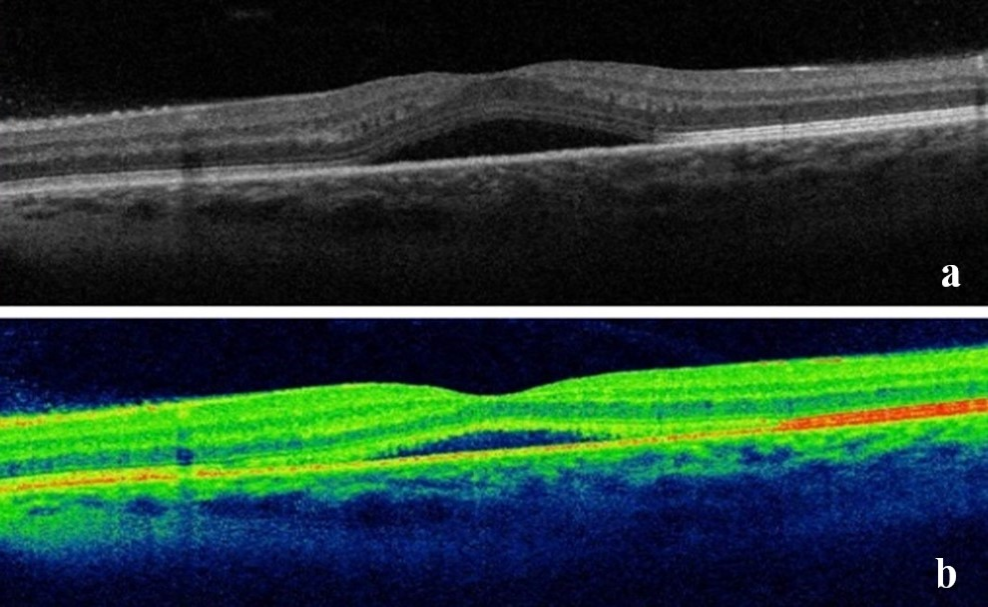
a) Central serous chorioretinopathy after paclitaxel; b) resolution of CSCR after one month

A 42-year-old female patient presented to our department with a one-week history of painless, subacute vision loss in her left eye. She had a history of hypothyroidism treated with levothyroxine (100 µg), rheumatoid arthritis treated with methotrexate (2.5 mg) and hidroxychloroquine (200 mg), and breast cancer with brain metastasis. Over five years, she was treated with neoajuvant high-dose cyclophosphamide, epirubicin and fluorouracil (3 cycles), neoadjuvant docetaxel and trastuzumab (3 cycles), modified radical mastectomy (T1cN1M0), adjuvant trastuzumab (17 cycles) and leuprorelin. After the diagnosis of brain metastasis she was treated with palliative radiotherapy (total cumulative dose: 50 Gy), paclitaxel, trastuzumab, and zolendronic acid started a year before the onset of the complaints. Her best corrected visual acuity (BCVA) was 20/20 in the right eye and 20/32 in the left eye. The anterior segment of both eyes was unremarkable. Intraocular pressure was normal. Fundus examination revealed macular edema and absent foveal reflex in her left eye. Optical coherence tomography (OCT) of the left eye showed central serous chorioretinopathy (CSCR) (Figure 1 (Fig. 1)) with spontaneous resolution within 1 month.

**Figure 2:**
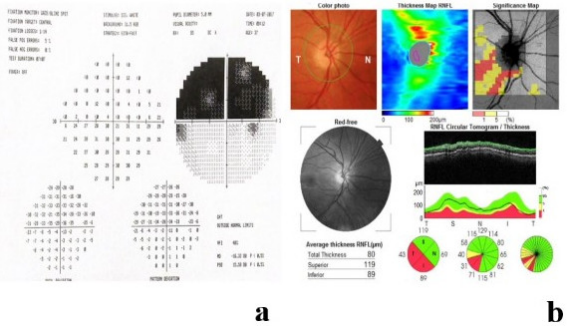
a) Altitudinal visual field defect of the right eye; b) optic disc pallor and RNFL thinning (temporal and inferior)

Seven months later, she presented with sudden decrease in vision in her right eye. The BCVA was 20/40 in the right eye and 20/25 in the left eye. The anterior segment exam findings and intraocular pressure (IOP) were in normal limits. The relative afferent pupillary defect (RAPD) was found in her right eye. Fundus examimation illustrated soft exudates in her left eye, supported by the OCT results. The Humphrey visual field of the right eye demonstrated a superior altitudinal defect corresponding to the inferior segmental optic nerve pallor (Figure 2 (Fig. 2)). 

**Figure 3:**
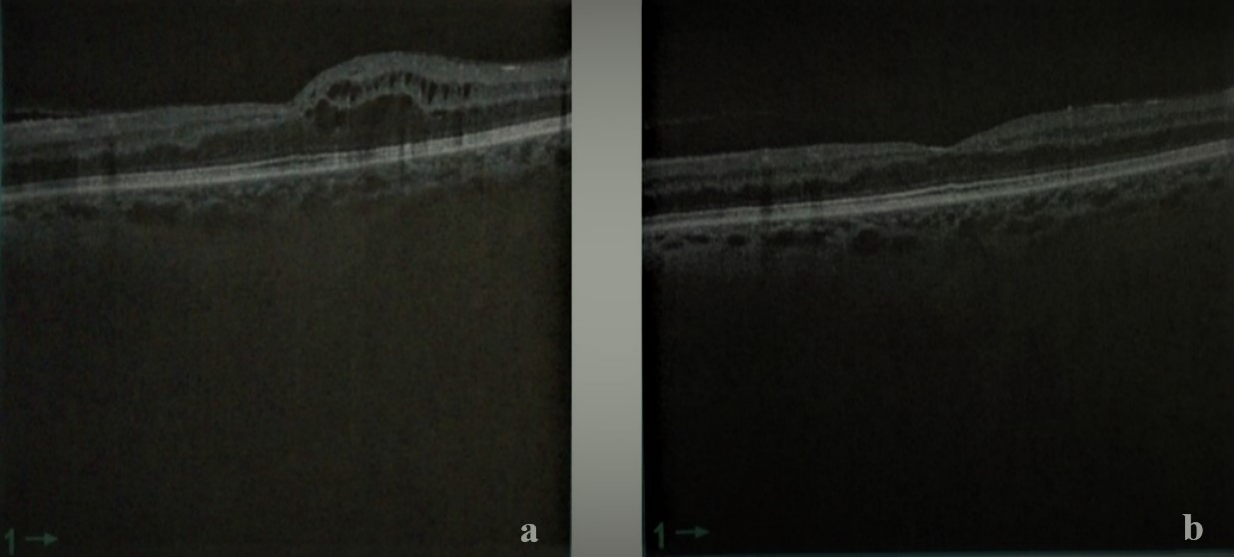
a) Macular edema before aflibercept injection; b) resorption of macular edema after aflibercept injection (one month later)

We started the treatment with prednisolone (50 mg/day) for one week, and the neurologist discussed hyperbaric oxygen therapy (HBOT) as an option for the RION with the patient. The patient refused HBOT and remained under follow-up. Six months later, her vision had fallen to light perception in the right eye and 20/25 in the left eye. The OCT findings were conclusive for cystoid macular edema (CME) in her left eye with a central macular thickness of 309 µm. We administered an intravitreal injection of aflibercept 40 mg/ml. The resorption of macular edema was observed at the one-month follow-up (Figure 3 (Fig. 3)); the BCVA was 20/160 in her left eye.

Unfortunately we could not improve the visual outcome in the right eye, which had an amaurotic pupil. Regarding the left eye, the patient experienced recurrent macular edema treated with aflibercept. After several episodes, the patient’s visual acuity decreased to 20/50 without any improvement after aflibercept injection.

## Discussion

Systemic chemotherapy and radiotherapy can be highly effective in maximizing therapeutic outcomes, particularly when used in combination [1], [5]. These treatments have improved overall survival and enhanced symptom management for patients with metastatic breast cancer, despite challenges such as side effects and drug resistance [5]. Ocular toxicity linked to chemotherapy and radiation is rare and may be under-reported, yet it remains a significant concern in maintaining the quality of life for oncology patients [3], [4], [6]. Our patient was initially diagnosed with breast cancer in 2011 and later developed brain metastases in 2016, for which she underwent chemotherapy with various agents and received radiotherapy. While her life expectancy improved, she experienced ocular side effects that significantly impacted her quality of life.

In combined chemotherapeutic treatments, identifying the specific agent responsible for a particular ocular adverse effect is challenging, leading to under-reporting of these side effects [7], [8]. Docetaxel and paclitaxel belong to the taxane family and are utilized as both first-line and second-line anticancer drugs in breast cancer treatment [8]. Paclitaxel-associated cystoid macular edema was reported in one patient (0.5%) in the study by Kaya et al. [9]. The pathophysiology of macular edema remains uncertain, with theories suggesting direct toxicity to Muller cells or the retinal pigment epithelium (RPE), or leakage of molecules smaller than fluorescein [10], [11], [12]. Impaired visual acuity is the main symptom, and in most cases, taxane-associated macular edema is bilateral and resolves spontaneously after discontinuing the medication [6]. In our case, the patient initially experienced unilateral CSCR, followed by CME in the same eye after 13 months. Central serous chorioretinopathy resolved spontaneously within 1 month, while CME was treated with intravitreal aflibercept and showed resolution one month later. However, the patient had multiple episodes of macular edema that were successfully managed with aflibercept until visual acuity did not improve further after treatment.

Trastuzumab is a monoclonal antibody used in the treatment of breast cancer by targeting and binding to HER2 receptors on cancer cells. It is commonly used alongside chemotherapy or other treatments for HER2-positive breast cancer and has significantly improved outcomes for patients with this subtype [13]. Trastuzumab can rarely cause macular edema and ischemic maculopathy, which may lead to substantial visual impairment. Saleh et al. [14] documented a case of bilateral ischemic maculopathy characterized by macular edema, retinal hemorrhages, and hard exudates. Ocular toxicity associated with trastuzumab treatment typically improves upon discontinuation of the medication but may recur if re-administration is attempted. The exact mechanism of this side effect remains unclear [14]. Our patient underwent both neoadjuvant and adjuvant treatment involving multiple cycles of trastuzumab, which may have contributed to the development of retinopathy, given trastuzumab’s association with retinal ischemia [15]. The patient’s vulnerability to this condition was probably increased by the combination of different chemotherapy drugs.

Paclitaxel-induced retinopathy may be exacerbated by concurrent long-term use of hydroxychloroquine for the treatment of rheumatoid arthritis, as demonstrated in the case reported by Elhusseiny et al. [16]. Hydroxychloroquine is widely used for treating rheumatic diseases and is generally well tolerated, although retinopathy may occur, and the damage can be subclinical. Based on dosage and treatment duration, hydroxychloroquine is associated with various forms of retinopathy, including both leaking and non-leaking CME, with 12 reported cases [17], [18]. The mechanism underlying hydroxychloroquine-induced CME remains unclear, but it is hypothesized to involve increased permeability of the RPE and impairment of its pumping function [18], [19], [20]. Our patient received long-term treatment with hydroxychloroquine for rheumatoid arthritis without developing photoreceptor damage, bull’s eye maculopathy via RPE damage, or diffuse retinal atrophy, as reported in other cases [21], [22]. However, taxanes could potentiate the non-leaking CME effect of other agents like hydroxychloroquine at the level of the RPE pump, as suggested by Buffet et al. [23], indicating a synergistic effect between these two therapies [16].

Radiation-induced optic neuropathy is a rare complication characterized by delayed radionecrosis affecting the anterior visual pathways, typically resulting from a cumulative radiation dose exceeding 50 Gy [24], [25], [26]. It causes severe visual acuity loss or reduction, which can occur in one or both eyes and is often irreversible [4]. Concomitant chemotherapy has been identified as a potential risk factor for RION [27]. The treatment for RION remains contentious; while many studies advocate for HBOT, corticosteroids, anticoagulants, angiotensin-converting enzyme inhibitors, and bevacizumab; their efficacy in treating RION has not been substantiated [28], [29].

A multidisciplinary approach is essential for treating metastatic breast cancer patients, particularly when considering the complex relationship between systemic therapies and ocular health. Collaboration between oncologists and ophthalmologists is crucial in monitoring and managing ocular side effects caused by chemotherapy and radiotherapy [8], [15]. 

## Conclusion

Herein we stated a young patient with metastatic breast cancer who developed a rare and unusual overlap of side effects: paclitaxel-associated retinopathy and radiation-induced optic neuropathy. We aim to illustrate the challenge of managing advanced breast cancer patients and emphasize the importance of careful monitoring for ocular complications, which can impact the patient’s quality of life.

## Notes

### Authors’ ORCIDs

- Suher Abduraman: 0000-0001-6128-2185
- Ali Riza Cenk Celebi: 0000-0002-7952-1241

### Authors’ contributions

Conceptualization: S.A., B.M. and A.R.C.C.; methodology: S.A., B.M. and A.R.C.C.; investigation: S.A. and A.R.C.C.; resources: S.A.; writing – original draft preparation: S.A.; writing – review and editing: B.M. and A.R.C.C.; supervision: A.R.C.C.

### Ethics statement

This study was approved by Acibadem University Ethical Committee and adhered to the Declaration of Helsinki.

### Informed consent

Written informed consent was obtained from the patient for publication of this case report and accompanying images.

### Data availability

Data supporting the study results can be provided followed by request sent to the corresponding author’s e-mail.

### Competing interests

The authors declare that they have no competing interests.
